# P-1562. Outcomes of Human Babesiosis and Spleen Abnormalities

**DOI:** 10.1093/ofid/ofaf695.1742

**Published:** 2026-01-11

**Authors:** Rudline G Zamor, Brigitte Maczaj, Victoria A Bateman, Abdullah Khan Zada, Akif Acay, Luis A Marcos

**Affiliations:** Stony Brook University Hospital, Stony Brook, NY; Stony Brook Medicine, Stony Brook, New York; Stony Brook University, Stony Brook, New York; Stony Brook University Hospital, Stony Brook, NY; Northport VAMC, Northport, NY; Renaissance School of Medicine at Stony Brook University, Stony Brook, New York

## Abstract

**Background:**

*Babesia microti*, a parasitic blood-borne piroplasm, causes the disease Babesiosis in humans. Outcomes of babesiosis and splenic abnormalities are limited. The aim of this study is to assess risk factors associated with splenic abnormalities in adult patients with Babesia infection.

**Table 1.**
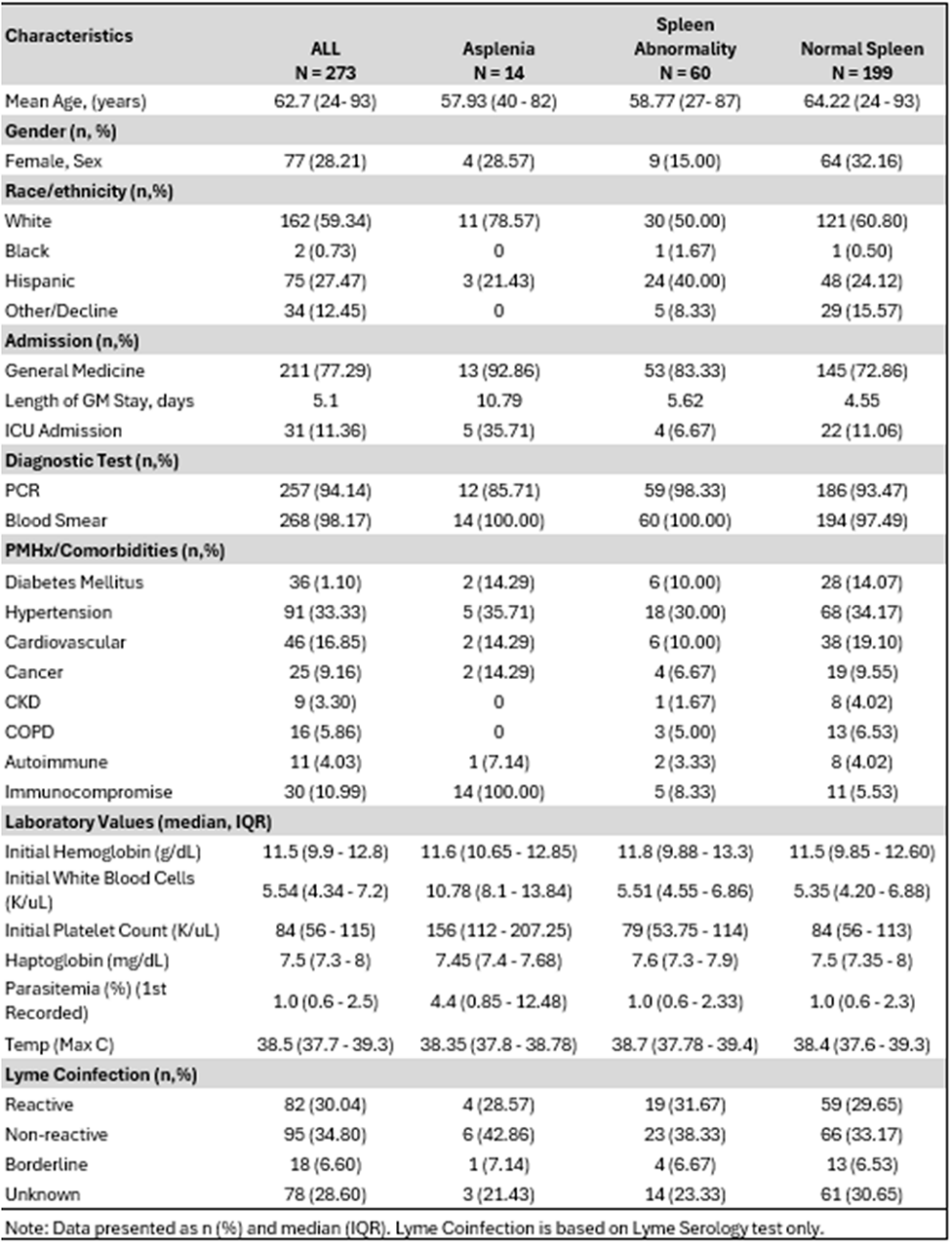
Descriptive table of patients’ characteristics by spleen presentation

**Table 2.**
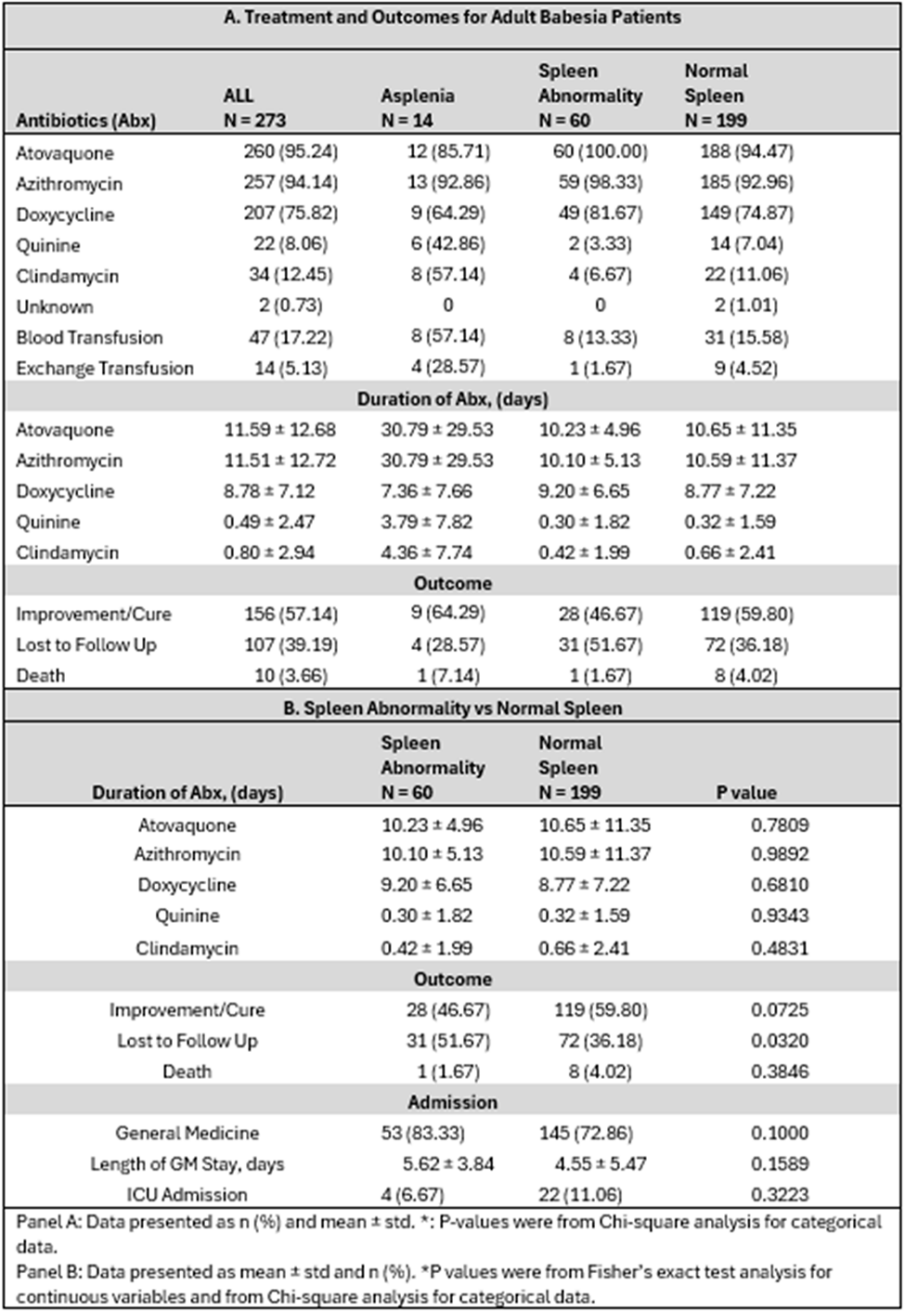
Treatment and Outcomes of Babesia Patients and Spleen Comparison

**Methods:**

Adult patients with peripheral blood smear positive for Babesia spp (confirmed by PCR) diagnosed at Stony Brook University Hospital between 2014 – 2024 were included in this study. Demographics, laboratory values, treatment, imaging and outcomes were collected by detailed chart review. Patients were grouped by status of spleen presentation: asplenia, spleen abnormality (infarct and/or enlargement) and normal spleen (normal size). Statistical analysis applied to categorical variables was by X^2^ and continuous variables was by Student’s *t* test. A p-value of less than 0.05 was considered statistically significant.

**Table 3.**
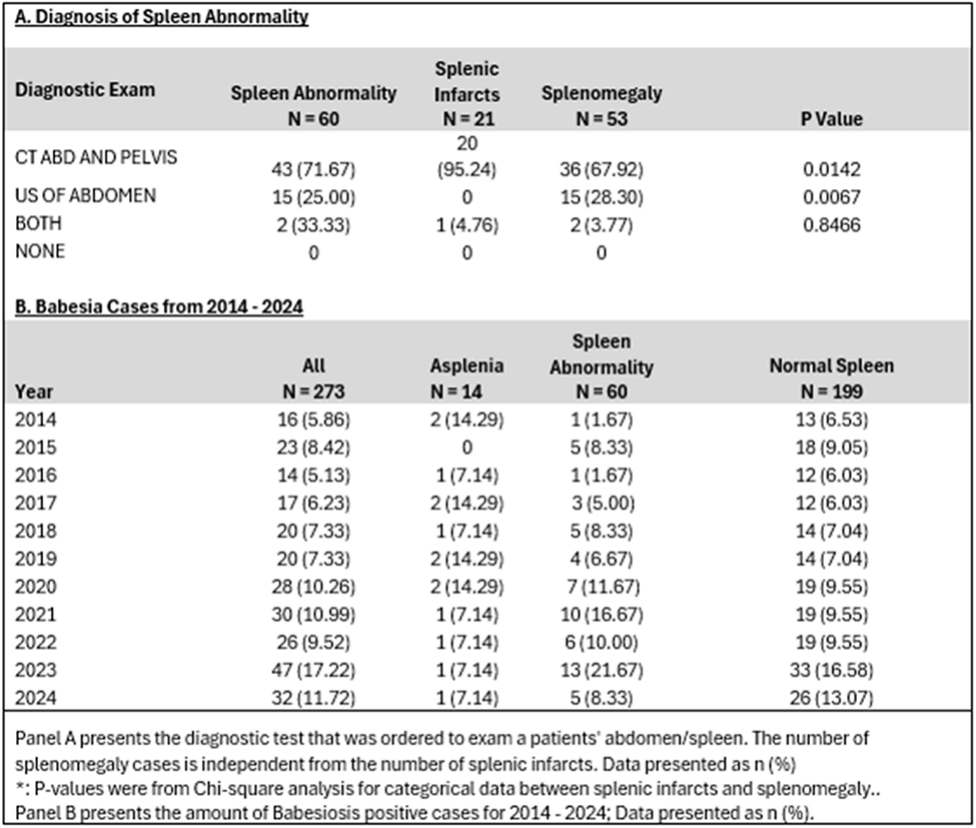
Descriptive table of spleen abnormalities and amount of Babesia cases, 2014 - 2024

**Results:**

A total of 273 adult patients (28.21% female) were identified with B*. microti* infection, 14 patients with asplenia, 60 with spleen abnormalities and 199 with normal spleen (Table 1). The average duration of atovaquone/azithromycin in days was found highest in asplenic patients (30.79 ± 29.53 days) than normal spleen patients (10.65 ± 11.35) (p=< 0.0001). Asplenic patients also had the longest hospitalization (10.79 days), exchange transfusion (28%) and ICU admission (35%). General admission was not found to be significantly different between normal spleen and spleen abnormality, (p=0.1000) (Table 2). Majority of splenic abnormalities were diagnosis through CT Abdomen and Pelvic exam (71.67%). Of the splenic abnormalities, there were 21 spleen infarcts and 53 splenomegalies; these cases were independent of the total amount the splenic abnormalities.

**Conclusion:**

Asplenia patients are shown to have longer hospitalization with prolonged antibiotics regimen, higher ICU admission and exchange transfusions than patients with spleen abnormalities and normal spleen. Patients with normal spleen had more mortality and more ICU admission than those with splenomegaly/infarcted.

**Disclosures:**

All Authors: No reported disclosures

